# “The Dose Makes the Poison”: Relevance of Paracelsus’s Principle for Modern Chemical Hazard Assessment with New Approach Methodologies

**DOI:** 10.1021/acs.est.5c14563

**Published:** 2026-01-14

**Authors:** Beate I. Escher

**Affiliations:** † Department of Cell Toxicology, 28342 Helmholtz Centre for Environmental Research − UFZ, Permoser Str. 15, 04318 Leipzig, Germany; ‡ Environmental Toxicology, Department of Geosciences, Eberhard Karls University Tübingen, Schnarrenberger Str. 94-96, 72076, Tübingen, Germany; # German Center for Child and Adolescent Health (DZKJ), partner site Leipzig/Dresden, Leipzig 04103, Germany

**Keywords:** Hazard, green toxicology, safe and sustainable by design, New Approach Methodologies, in vitro bioassay, persistence, baseline toxicity, adverse outcome pathway

## Abstract

New approach methodologies (NAMs) including in vitro assays and in silico prediction methods that are based on mechanistic understanding of the pathways of toxicity have changed how we nowadays assess the hazard and risk of chemicals in commerce. Quantitative dose–response relationships obtained from NAMs are often dichotomized into categorical outcomes (toxic/nontoxic), introducing uncertainty and potential misclassification in hazard characterization because the applicability domain of NAMs is currently constrained to the medium-hydrophobicity segment of the chemical universe. Until physicochemical limitations to the testability of (super)­hydrophobic chemicals are overcome and hydrophilic and charged chemicals are tested at higher doses than is present practice, “no response” should not be equated with an absence of toxicity. Toxicity is not categorical—it is the dose that makes the poison. Data gaps for difficult-to-test chemicals can be filled with baseline toxicity predictions. Baseline toxicity is the minimum toxicity, but it is often a robust predictor. Persistence constitutes a hazard criterion in its own right. Combining persistence with toxicity into a “persistent toxicity” metric may provide a more integrative indicator of the hazard potential. Persistent toxicity indicators measured with NAMs could play a pivotal role in comparative hazard assessment for substitution chemicals and the development of safe and sustainable by design (SSbD) chemicals.

## Graphical abstract

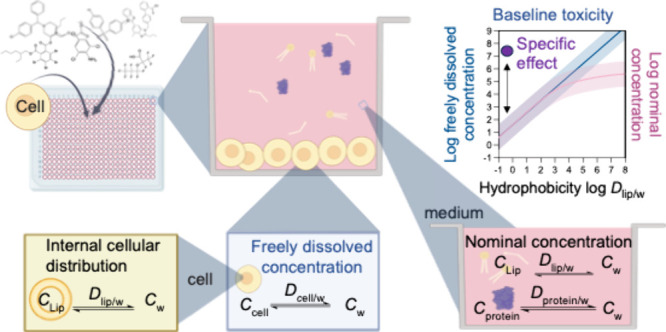

## Introduction

Next generation hazard and risk assessment is moving away from animal-based testing to so-called “New Approach Methodologies” (NAMs), which encompass in vitro and in silico methods.^1,2^ There is hope that this development not only saves millions of test animals but also is a means to cope with the exponentially increasing number and diversity of chemicals on the market^3,4^ that has led to ubiquitous contamination of the environment, biota, and humans with complex mixtures of chemicals.^5,6^

In vitro NAMs can be based on human, animal, and plant cells and can be applied for human health and environmental hazard and risk assessment. They yield fundamental mechanistic information about the toxicity pathway. In the case of human hazard assessment, NAMs avoid the need to extrapolate from animals to humans. NAMs will play an important role in the “Defined Approach” (DA), as already outlined for the example of skin sensitization, in that “results from multiple information sources can be used together in DAs to achieve an equivalent or better predictive capacity than that of the animal tests to predict responses in humans”.^7^

For developing chemicals that are “safe and sustainable by design” (SSbD)^8^ we need simple hazard indicators that can be predicted prior to synthesizing new molecules. Fenner and Scheringer^9^ argued the case that “chemical principles in environmental chemistry and toxicology [are] still underexploited under the current testing paradigm” and that chemical simplification is the only way out of the dilemma of increasing numbers and diversity of chemicals without proper hazard information and that chemicals have to be grouped in classes and jointly assessed. Muellers et al.^10^ recently proposed property-based chemical regulation supported by the recognition that similar/shared properties will lead to similar hazards. This would lead immediately to a reduction of regrettable substitutions, which occur when replacement products for phased-out chemicals turn out to be as problematic as the original banned chemical. Unfortunately, at present, chemicals with small changes of the chemical structure are initially presumed innocent despite their structural similarity with a hazardous chemical, which has led to the explosion of untested or inadequately tested chemicals, rendering risk assessment unmanageable.^6^ Since perfluorooctanoic acid (PFOA) and perfluorooctanesulfonic acid (PFOS) were restricted, thousands of replacement products have flooded the market and ultimately the environment.^11^ Comparative hazard assessment approaches using NAMs could close these loopholes of current chemical legislation such the European REACH regulation,^12^ which is in its present form vulnerable to regrettable substitutions. Furthermore, the combination of persistence and toxicity assessment into one indicator, “persistent toxicity”, may further strengthen hazard assessment.

The following analysis begins with a discussion of the importance of viewing toxicity as a gradual phenomenon rather than a binary one. It also discusses how current interpretations of NAMs are limited by their restricted coverage of physicochemical space. This is particularly problematic for highly hydrophobic chemicals, which are often considered nontoxic because they cannot be easily tested. Next, there is a discussion of the differences among pharmaceuticals, pesticides, and industrial chemicals, as well as what these differences mean for their testing with NAMs. Currently, the focus is on the biological perspective of NAMs, but this should be complemented by a chemical perspective. This requires considering dose metrics and comparing the measured effects to baseline toxicity predictions in order to identify specific and reactive toxicity. Then, the application of NAMs for the development of toxicity prediction models and comparative hazard assessment are discussed. A new proposal is to expand NAMs from toxicity assessment to a “persistent toxicity” assessment by coupling high-throughput biodegradation testing with toxicity testing.

## The Dose Makes the Poison

Toxicity refers to the ability of a chemical to cause harmful effects. There is no such thing as “not toxic”. Paracelsus, the great physician and philosopher of the Renaissance (16th century), stated: “What is there that is not poison? All things are poison, and nothing is without poison. Solely the dose determines that a thing is not a poison.” ^13,14^ Unfortunately, many scientific papers and prediction models consider toxicity as categorial (toxic/nontoxic, mode of action present/not present). This is misleading because if the response of a chemical is “no effect detected at the highest tested dose”, it is often erroneously reported as nontoxic.

There are two ways to present toxicity data from NAMs that were measured with a series of increasing dose or concentration: (i) as concentration–response curves, described by an equation from a best fit regression model, from which an effect concentration with a defined effect (e.g., 10% cytotoxicity, *x*% activation of a receptor) can be derived, or (ii) as no observed effect concentration (NOEC) and lowest observed effect concentration (LOEC), which are derived by statistical hypothesis testing of every concentration level as compared to an unexposed control. Especially in ecotoxicology, there is a controversy about which parameter is better.^15–17^ Without making any judgment as to which way is better, one needs to be mindful that NOECs are often erroneously mistaken as truly no effect. In contrast, most concentration–effect models, among which the most popular is the log-sigmoidal concentration–effect relationship,^18^ approach zero only asymptotically, which means that a true zero effect is achieved only at zero concentration,^19^ aligning with Paracelsus’ principle.

The paradigm of threshold effects in toxicology (with exception of cancer, where it is accepted that there is no threshold) has led to the widespread belief that a chemical will not only be inactive below concentrations of the measured effect threshold but also not contribute to mixture effects.^20^ This is incorrect because theoretical considerations^21^ and numerous experimental studies^22–24^ have shown that chemicals present below their individual effect thresholds can contribute to mixture effects. Therefore, no chemicals should be exempted from hazard assessment a priori.

In many standard toxicological studies using NAMs, e.g., in the large-scale testing of thousands of chemicals in ToxCast and Tox21,^25^ the maximum dosed (nominal) concentration in in vitro assays was around 100 μM and dilution series with 15-point, half-log serial dilution typically extended down to 46 nM. Any chemical that was not active or toxic up to that concentration was considered “not toxic”. This is not correct; while very hydrophilic chemicals indeed need higher nominal concentrations than typically dosed, often in the millimolar concentration range, to cause a measurable effect, they cannot be dismissed as nontoxic. On the other end of the hydrophobicity spectrum, superhydrophobic chemicals are very difficult to dissolve and may have slow uptake kinetics so that they may not build up high enough concentrations in a cell during the time scale of the assay or form invisible microcrystals in the medium. The same problem persists for in vivo aquatic toxicity testing, but at least in fish toxicity assays the dissolved concentration is monitored routinely.^26^

## Intentionally and Not Intentionally Potent Chemicals

Scheringer and Schulz^6^ proposed to distinguish industrial chemicals from pesticides, biocides, and pharmaceuticals. The latter are “intentionally potent chemicals”, whose use must be authorized. Not only the efficacy of their intended use but also their environmental and human health risk must be assessed. Consequently, their numbers are much smaller than that of the entire chemical universe, with approximately 4000 active ingredients in pesticides and biocides and 3000 pharmaceuticals worldwide.^6^ All of them cause highly specific target effects, be it beneficial for pharmaceuticals or adverse for pesticides. The question that needs to be answered for them is whether there are also off-target effects. Such chemicals will always forego the hazard classification step as they are hazardous per definition, but move straight to risk assessment.

In contrast, industrial chemicals that are “not intentionally potent” are developed for functions unrelated to biological potency, yet they may still elicit biological effects unintentionally. It is estimated that >300,000 chemicals fall under this category worldwide, and approximately 30,000 industrial chemicals are registered in Europe under REACH.^6^ While 350,000 chemicals are in the Classification and Labeling (C&L) Inventory of the European Union, there are only 4,400 EU-level harmonized classifications, which means that less than 1% of the classifications are legally binding.^27^ Only a fraction of all industrial chemicals have experimental toxicity data.

For industrial chemicals, it is essential that hazard assessment is fast, reliable, and comprehensive. Even if NAMs are used to accelerate the hazard assessment, they must not be too complex but simple, accessible, robust, and high-throughput. The likelihood that one industrial chemical has only one highly selective effect on one toxicity pathway is low. Rather, one would expect that several pathways are activated/inhibited and that the intrinsic potency is not very high. This shifts the focus from the present ambition to make NAMs as biologically realistic as possible and identify the most subtle effect to building a battery of high-throughput NAMs that covers broadly possible interactions between chemicals and biomolecules in cells. What further complicates the assessment of industrial chemicals is that many are (super)­hydrophobic. Pharmaceuticals and most modern pesticides are of medium hydrophobicity, so that they can be easily absorbed by the target, while some industrial chemicals such as brominated flame retardants or UV-absorbers are so hydrophobic that they are not testable with current methods, as will be discussed in detail below.

## The Biological Perspective of NAMs

The term NAM was introduced in 2016 during a workshop of the European Chemicals Agency (ECHA).^28^ NAMs include in silico approaches and in chemico and in vitro assays, and their purpose is to improve read-across, prioritization, and screening in regulatory chemical hazard assessment for human and environmental health. One of the key conclusions was that “NAMs have reduced uncertainty in toxicodynamics and could help confirm mechanism of action”.^28^ The workshop participants agreed on the goal of full replacement of animal testing in risk assessment in some areas.

In the decade since this workshop, NAMs have taken off, moving from mechanistic understanding of chemicals’ mode of action to the goal of full replacement of animal testing in risk assessment.^4^ There is a common understanding that NAMs need to be solidly rooted in adverse outcome pathways (AOPs).^29^ AOPs describe the network from molecular initiating events (MIEs) over key events (KEs) to adverse outcomes (AOs). Especially MIEs and KEs that are connected to multiple AOs should be prioritized,^30^ potentially also connecting human and environmental hazard assessment with the same NAMs.^31^ Proposed test systems have evolved from the initially simple high-throughput screening tools to very sophisticated stem cell,^32^ organoid^33,34^ and organ-on-a-chip models,^35^ often in combination with transcriptomics.^36^

How complex must an in vitro assay be? Is there a trade-off between biological realism vs testability of wide range of chemicals? This depends on the purposes: For research and risk assessment, biological realism is of utmost important. For hazard assessment and design of substitute chemicals, priority should be given to bioassays that are reliable, scalable, and quantitative—that is, they can be run reproducibly across many samples, handle thousands of chemicals efficiently, and provide numeric results suitable for comparing the relative hazards of substances.

The more complex the in vitro assay, the more difficult are comparisons because dose–response relationship are obscured by variability in bioavailability, requiring efforts to measure or model in vitro disposition,^37^ while in practice, the majority of NAMs results are reported as nominal concentrations as discussed in more detail in the section Biologically Effective Dose and Other Dose Metrics.

The “EU Partnership for the Assessment of Risk from Chemicals” (PARC) is in the process of collating NAM assay batteries^38^ for developmental (DNT) and adult neurotoxicity (ANT),^39^ thyroid hormone disruption,^40^ metabolic endocrine disruption,^41^ nongenotoxic carcinogenicity,^42^ and immunotoxicity.^43^ The DNT test battery is most advanced with 120 chemicals tested in 10 assays, and a further 12 assays are identified for future expansion.^44^ Again, one has to differentiate between intentionally and unintentionally toxic chemicals. For instance, the evaluation of developmental toxicity of pharmaceuticals will require much more in-depth assessment^45^ than hazard ranking of substitution chemicals of phased-out chemicals.

These test batteries are important for a comprehensive toxicological assessment and mechanistic evaluation, but for hazard assessment, they need to be simplified and the number of assays must be reduced to be able to cover thousands of chemicals, while assuring that both human and ecotoxicological end points are covered (Figure 1).

**Figure 1:**
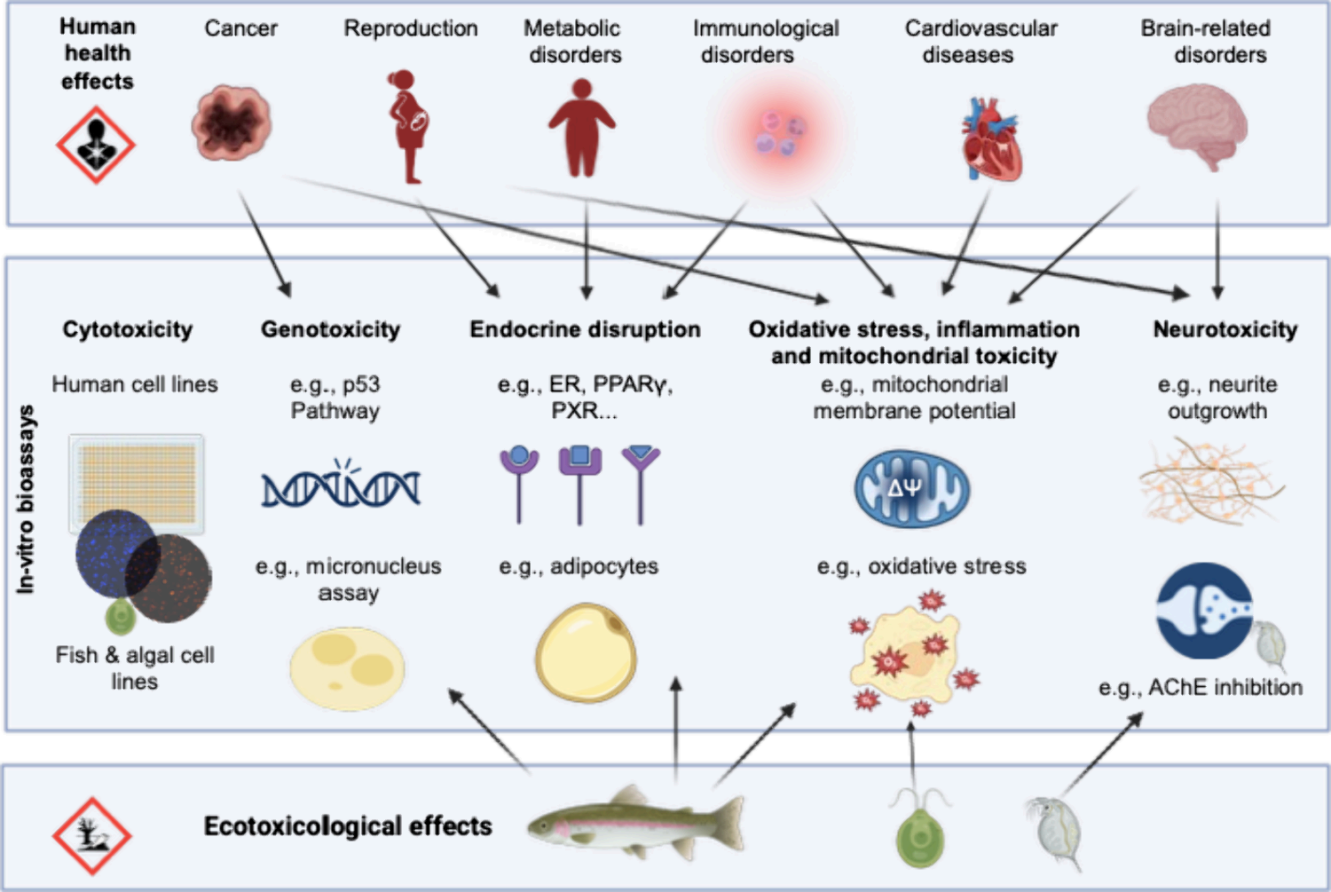
In vitro bioassays that connect human health and ecotoxicological effects. The arrows are not comprehensive but only point out the most common connections. Figure partially created in BioRender. Escher, B. (2025) https://BioRender.com/z7fef86.

Many pathways are highly conserved in vertebrates and invertebrates, and many MIEs and KEs are universal across cell types. A chemical that can damage DNA will do so in all cell types, but there are differences in defense mechanism that will modify the AO. A minimum test battery that covers the six clusters of human disease^46^ as well as ecotoxicological effects should include bioassays indicative of cytotoxicity and the sentinels of aging and stress, namely, oxidative stress, inflammation, and mitochondrial toxicity,^47–49^ plus assays indicative of genotoxicity, endocrine disruption, and neurotoxicity (Figure 1). For hazard assessment, select responsive assays will suffice, and while different cell types should be included, not all permutations of relevant MIE and KE with all sorts of cell types are necessary. For example, estrogenic effects are problematic for fish reproduction as much as for human health, but the principle is conserved, and one reporter gene assay based on the human estrogen receptor will also be indicative for such effects in other species.

## The Chemical Perspective of NAMs

What is missing in the discussion of the implementation of NAMs in chemical hazard assessment is the chemical perspective. As discussed above, industrial chemicals are not intentionally potent, but they cover a huge physicochemical property space. Many NAMs can accommodate only the testing of a limited diversity of chemicals due to the makeup of bioassays and have often undefined dosing conditions. This topic will be discussed in the next section; here, consideration is given to what types of MIEs are in principle possible and how they can be grouped from the chemical perspective.

The fundamental principle of the AOP is that one or more MIE(s) and KE(s) are conditional for the AO, but that the MIE will not invariably lead to the AO due to defense mechanisms or too weak effects on the MIE and KE level. For hazard assessment, the manifestation of MIE(s) and KE(s) should be sufficient provided that a causal relationship between MIE and AO has been established in principle even if not for the given chemical. One could further argue that it is not necessary to evaluate all possible MIE(s) and KE(s) as this would be overwhelming but to focus on how and how strongly a chemical interacts with a biological target to quantify its hazard.

Only a limited number of interactions of chemicals with biomolecules exist. The consequences differ dependent on the cell type, end point measured, organ, and system, but the initial trigger will be similar across various assay systems. In ecotoxicology, there has been a consensus for decades that modes of action are essentially rooted in the interactions between the chemical and the three main types of biomaterials, membranes, proteins, and genetic material.^50^

Weak and reversible interactions include van der Waals interaction and weak hydrogen donor/acceptor interactions that lead to partitioning and nonspecific binding.^51^ Important examples are partitioning into biological membranes, e.g., cell membranes, nerve cells, or mitochondria. Such interactions cause reversible nonspecific effects but may still lead to ultimately irreversible effects. The effects of the same dose of chemical may be of varying potency depending on the functionality of the membrane, but there are commonalities between different targets for nonspecific effects. If we know that a surfactant or a surfactant-like PFOS disturbs membrane structure and functioning, it will do so in any membrane it reaches, but the adverse effect will manifest first in processes that are critical, e.g., energy transduction in mitochondria. If energy is depleted, many cellular and body functions are not possible anymore, and all these effects can be quantified, but to assess the hazard, it would be sufficient to quantify the initial membrane disruption or mitochondrial toxicity.

Binding to proteins, receptors, and enzymes may also be nonspecific or much stronger to almost irreversible due to steric fit in binding niches via van der Waals interactions and strong hydrogen bond donor/acceptor bonds. Such interaction with receptors and enzymes will be highly specific, but many receptors and enzymes are highly conserved across species with only subtle differences in their binding domains.

Formation of covalent bonds with biomolecules leads to membrane damage, protein damage and depletion, and DNA damage. Formation of reactive oxygen species and oxidative stress can also be triggered by reactive chemicals. At the same time, very reactive chemicals are often not even stable enough to be taken up into the cell but already undergo some abiotic degradation in the bioassay system. Other chemicals will be activated only by metabolism inside the cell and organism to a reactive form, catalyzed by metabolic enzymes in cells and particularly in liver tissue.

Many of these interactions are uniform across all cells, but not every cell in the human body is the same. Selective toxicity refers to effects that are occurring in certain cell types, organs, or—if we expand to ecotoxicology—species. The feature of selective toxicity is exploited in the development of pesticides that should kill the pest but no other species or pharmaceuticals that should target one specific pathway but not cause off-target effects. Let us remember these are intentionally potent chemicals and their safety assessment is much more sophisticated than for industrial chemicals that are not intentionally potent. Therefore, the likelihood of high selectivity is low among industrial chemicals, and the search for selectivity does not need to be prioritized for hazard assessment. There are exceptions, for instance, in the case of industrial chemical groups where the functionality may be directly or indirectly connected to toxicity. An example is antioxidants and antiozonants. As part of their function, they capture reactive oxygen species but form often problematic transformation products in this process, e.g., phenolic antioxidants may form carcinogenic quinones^52^ or the tire-rubber antiozonant 6-PPD formed a very potent ichthyotoxin (6PPD-quinone) in surface water.^53^

In ecotoxicology, before the advent of AOPs, chemicals were classified into ten groups of modes of action according to their target sites and type of interaction with the biomolecule.^50^ While this view might be too simplistic for human health effects, I posit nevertheless that a test battery of NAMs should cover all types of interactions and include a manageable number of bioassays to ensure that hundreds and thousands of chemicals can be screened (Figure 1). A chemical is hazardous if it has high baseline toxicity and/or if one or more targets are affected with high specificity. What is meant by “high specificity” is addressed in the next sections.

## Biologically Effective Dose and Other Dose Metrics

The biologically effective dose is the concentration at the target site in the cell, e.g., membranes, cytoplasm, proteins.^54^ This target concentration is not experimentally available. The next best proxy is the freely dissolved concentration in the assay system because it can be directly compared between different assay types that use different formats and media, and it can be assumed that in steady state the freely dissolved concentration in the medium is the same as in the cytosol. While measured dissolved concentrations are standard in aquatic toxicity testing, and there are experimental methods to measure them even in 384-well plates,^55–58^ the dose in in vitro assays is typically reported as nominal concentration, i.e., the amount of chemical added to the cells. Various processes have to be considered when chemicals are dosed to cellular assays in well plates, many of them lowering the freely dissolved and hence bioavailable concentration (Figure 2a): a fraction of chemicals may bind to lipids or proteins in the medium or to the plastic well plates. Loss due to partitioning to the air space is also possible (not shown in Figure 2a). Only a fraction of dosed chemical is taken up by the cells. There exist numerous equilibrium partitioning models to predict the exposure of neutral medium-hydrophobic compounds,^37,59^ and they have been extended to ionizable chemicals.^55^ However, these models are not applicable to (super)­hydrophobic chemicals yet. Slow uptake kinetics of very hydrophobic and some charged chemicals are likely to underestimate their toxic potential, but there is a lack of robust experimental data to prove the case.

**Figure 2:**
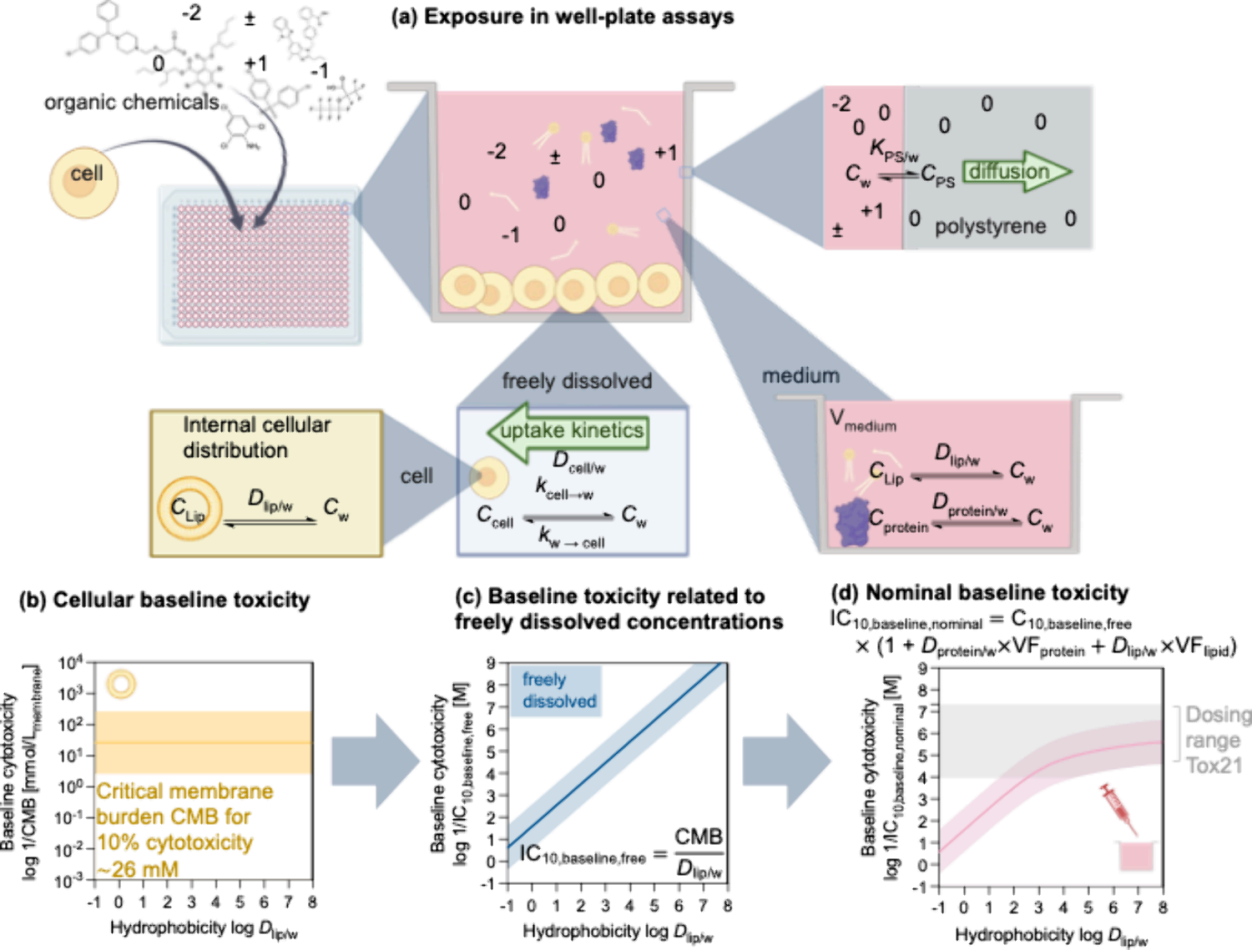
(a) Processes in well plate-based bioassays relevant to describe cellular concentrations.^55,65–67^ Medium–air partitioning is an important process for volatile chemicals but is not depicted. (b) The critical membrane burden (CMB) for baseline toxicants is independent of the chemicals’ hydrophobicity (expressed as liposome–water distribution ratio *D*_lip/w_). (c) Relationship between freely dissolved inhibitory concentration triggering 10% cytotoxicity IC_10,baseline,free_ and the chemicals’ hydrophobicity. (d) Same relationship for nominal baseline toxicity IC_10,baseline,nominal_ accounting for the binding to system components described in (a). *D*_protein/w_ = protein–water distribution ratio, VF_protein_ = volume fraction of proteins, and VF_lipid_ = volume fraction of lipids in the bioassay. Binding to well plate plastic is negligible if protein content in medium is sufficiently high.^64^ Figure 2 is somewhat simplified in that in drawing the plot (d), it was assumed that the protein binding is proportional to the distribution to membrane lipids. This is not the case for anionic chemicals, but the IC_10,baseline,nominal_ can still be reliably calculated with the equation given in Figure 2 if experimental protein binding data log*D*_protein/w_ are available. Figure partially created in BioRender. Escher, B. (2025) https://BioRender.com/k32br6s.

It will not be necessary to measure freely dissolved concentrations for all chemicals if one is only interested in hazard assessment, but the differences in sensitivity between different in vitro systems due to differences in lipid and protein content of medium and protein and other system parameters need to be accounted for in quantitative in vitro to in vivo extrapolation (QIVIVE)^60,61^ and for a comprehensive risk assessment.^62^ Several authors have argued that nominal concentrations are a conservative approximation in QIVIVE,^62,63^ but this only holds for chemicals with an octanol-partition constant log*K*_ow_ < 4, and increasing deviations of the freely dissolved from the nominal concentration are expected for more hydrophobic chemicals. For example, in a 96 well-plate format <1% of the dosed chrysene (log*K*_ow_ = 5.81) was freely dissolved as quantified by solid-phase microextraction.^58^

Intuitively one could then just lower the protein and lipid concentration in the bioassay medium to avoid binding to medium colloids, but this would lead to instable dosing conditions because hydrophobic chemicals do not like to be freely dissolved and will bind to anything, including plastic well plates; there may be residual sorptive medium components; and the cellular uptake will deplete the dosed concentration. In contrast, by keeping a defined protein and lipid content in the medium, one can stabilize the freely dissolved and cellular concentration. If the freely dissolved concentration is decreasing by cellular uptake or other loss processes, then it is replenished by desorption from the medium proteins and lipids. We call this serum-mediated passive dosing (SMPD).^64^ If uncontrolled, these factors can cause apparent sensitivity differences between bioassays that reflect the assay setup or medium composition rather than true potency differences.

## Anchoring in Baseline Toxicity

Nonspecific toxicity, also called baseline toxicity, is the minimal toxicity any chemical exhibits. Baseline toxicity is an important concept in aquatic toxicology^68^ and has for many decades been used to analyze the degree of specificity of effects.^69^ Baseline toxicity is triggered by chemicals intercalating and interacting with biological membranes and disturbing their structure and functioning.^70^ This effect is virtually independent of the chemical characteristics and only slightly dependent on molecular volume but occurs at constant critical membrane burdens (CMBs).^71–74^ Recently, attempts have been undertaken to define the CMB also for typically used cell-based bioassays. A combination of experiments and equilibrium partitioning models have set the CMB that triggers 10% cytotoxicity in diverse human cell lines to 26 mmol/L_membrane_ (Figure 2b).^75^ There is uncertainty and variability to this CMB by at least a factor of 10 in each direction, and we can expect further refinements in the future. For the time being, this CMB serves as a convenient anchor to evaluate NAM data. Reactive and specifically acting compounds are active at lower concentrations than the baseline toxicity. By comparing predictions for baseline toxicity with experimental cytotoxicity or activity data it is possible to obtain a first impression if a chemical acts specifically.^76^

Baseline toxicity does not equal low toxicity, as many erroneously believe. This misconception of low or even negligible baseline toxicity has hampered the uptake of this concept in human toxicology and the field of NAMs. Hydrophobic chemicals have a baseline toxicity (lower effect concentration) higher than that of hydrophilic chemicals. This is visualized in Figure 2c, where the freely dissolved baseline cytotoxicity concentrations triggering 10% cytotoxicity (inhibitory concentration IC_10_) are calculated by dividing the CMB through the liposome–water distribution ratio *D*_lip/w_.^75^ The *D*_lip/w_ is used instead of the more commonly applied *K*_ow_ to include also organic chemicals with complex speciation and multiple charges. At present, there is limited data available for chemicals with log*D*_lip/w_ > 8, but apart from kinetic limitations there is no reason to believe that this relationship does not continue for more hydrophobic chemicals. If IC_10,baseline,free_ is converted to IC_10,baseline,nominal_ by a simple equilibrium partitioning model (Figure 2d),^75^ the IC_10,baseline,nominal_ overlaps with IC_10,baseline,free_ for log*D*_lip/w_ ≤ 3, but at higher log*D*_lip/w_ the correlation between IC_10,baseline,nominal_ and hydrophobicity starts to level off. This is because hydrophobic chemicals bind strongly to the proteins and lipids of the medium, and the fraction freely dissolved is low. As discussed above, lowering the serum content is not a solution, because it leads to very unstable dosing conditions and additional loss processes. It is recommended to rely on SMPD but to account for the lower apparent sensitivity due to binding to medium colloids by applying the equilibrium partitioning model presented in Figure 2d.

## Are (Super)Hydrophobic Chemicals Nontoxic?

(Super)­hydrophobic chemicals (log*K*_ow_ > 6) often appear to be inactive in typical cellular bioassays up to the highest tested concentration. However, “no response” does not mean “not toxic” but rather “likely very toxic but standard test systems cannot quantify the potency”. There is already a provision for this in the CLP, where the guidance document states “Lower quality information showing no or low toxicity should specifically be treated with care, especially where the quality assessment has revealed points of concern regarding methodology and reporting (e.g. maintenance of test concentrations). In addition, it should be noted that substances which are difficult to test may yield apparent results that are not indicating the true toxicity.”^77^

The dosing of superhydrophobic chemicals remains one of the greatest experimental challenges in the application of NAMs and animal toxicity testing but is also theoretically limited. The high melting points of many hydrophobic chemicals further reduce their aqueous and membrane solubility so that theoretically, not enough chemical can be dosed to reach the CMB.^78^ Consequently, very hydrophobic chemicals yield responses only if their effect is highly specific or if their solubility is close to the solubility of the subcooled liquid, i.e., the melting point is low. This is why we can easily quantify the activation of the arylhydrocarbon receptor by dioxin-like chemicals that occurs at concentrations far below the CMB but not their cytotoxicity. In addition to this thermodynamic problem, hydrophobic chemicals have lower uptake rates and cells are typically growing during the exposure experiment of typically 24–48 h for HTS cell-based bioassays, which means that steady state might not be reached.^79^ Then cellular concentrations would vary from experiment to experiment and from assay to assay.

## Specific and Reactive Toxicity as an Evaluation Tool

Chemicals that are reactive or specifically acting will have IC_10_ lower than IC_10,baseline_. The degree of enhanced cytotoxicity can be quantified by the so-called toxic ratio (TR), which is the ratio between IC_10,baseline_ and IC_10_.^80^ In the case of NAMs, often another effect end point than (cyto)­toxicity is quantified, e.g., the activation of a hormone receptor or an adaptive stress response. The effect concentration is then expressed as relative activity concentrations, e.g., the activity concentration at cutoff (ACC) or the median activity concentration (AC50). For the specificity analysis outlined below, these toxicity values need to be converted in absolute 10% effect concentrations (EC_10_) and the specificity ratio (SR_cytotoxicity_) can be defined as the ratio IC_10_ for cytotoxicity to the EC_10_ for the specific effect, and SR_baseline_ is the same ratio with IC_10,baseline_.^76^ If the SR_baseline_ is close to 1, the effect is not specific but can be predicted by baseline toxicity.

In Figure 2d the problem of potentially too low dosing for chemicals with log*D*_lip/w_ < 3 also becomes apparent with the gray area covering the dosing range of Tox21/ToxCast assays. Unless they have a high TR or SR, these hydrophilic chemicals will not be considered as toxic or active because their activity zone falls outside the dosing range that is marked in Figure 2d as a gray box.

The problem of missing data due to the limited dosing range is illustrated in Figure 3 for the example of the ToxCast data set for one reporter gene assay, ARE-bla, that is indicative of the antioxidant response element signaling pathway (keap-NRF2-ARE).^81^ The cellular oxidative stress response is an important adaptive stress response and one of the key indicators of environmental stressors involved in numerous AOPs.^82^ Dose–response data were reanalyzed for several ToxCast assays and TR and SR were derived.^76^ In ARE-bla 85% of 6744 chemicals were not cytotoxic, and 15% were cytotoxic (Figure 3a). The noncytotoxic and cytotoxic chemicals ranged over 15 orders of magnitude in hydrophobicity as represented by the log*K*_ow_ (Figure 3b).^76^ If the 5756 chemicals without cytotoxicity response merely acted baseline toxic, their subset of 3843 chemicals with log*K*_ow_ < 3 would have been incorrectly assigned as “not cytotoxic” because they were not dosed high enough. The 198 “not cytotoxic” chemicals with log*K*_ow_ > 6 are also likely artifacts because such hydrophobic chemicals are always highly cytotoxic even if they are merely baseline toxicants unless they are rapidly degraded in the bioassay. Thus, ToxCast/Tox21 data are not suitable to be converted to categorical toxicity data but should only be considered for their high-quality dose-response data.

**Figure 3:**
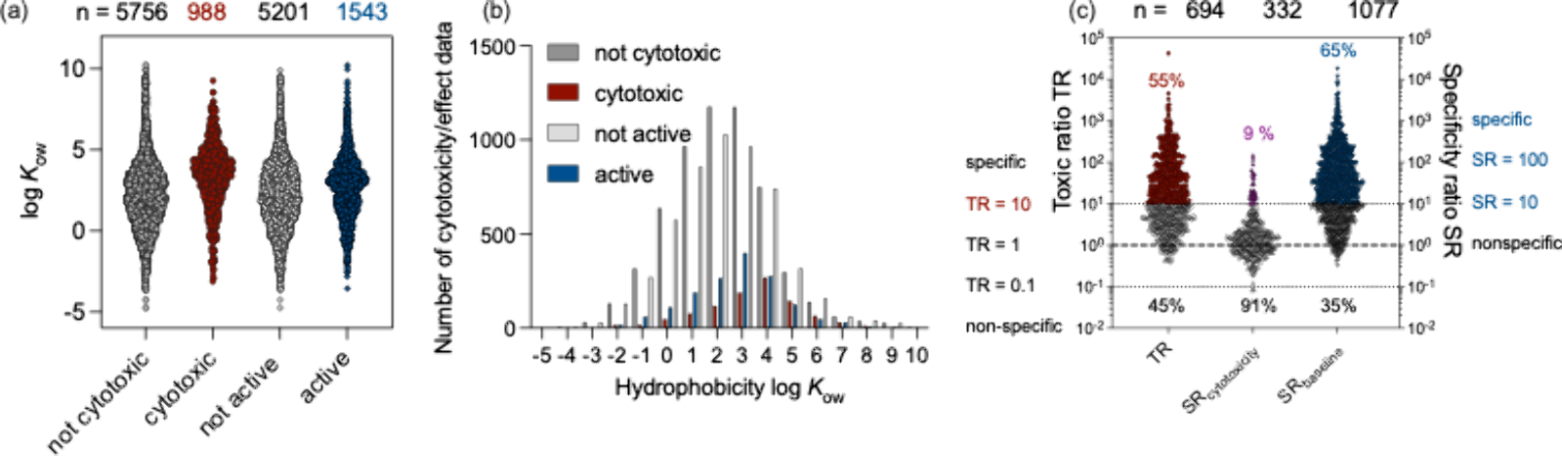
(a) Data reanalyzed from ToxCast data on ARE-bla by Escher et al.^76^ Only if the concentration–response curve exceeded 30% was the IC_10_ or EC_10_ concentration derived; otherwise, the chemical was classified as not cytotoxic or not active. Hence, the number of hits was lower than reported on the Chemistry Dashboard.^84^ (b) The same data set as histogram that binned according to hydrophobicity, expressed as octanol–water partition constant log*K*_ow_. (c) Toxic ratio (TR) and specificity ratio (SR) analysis of this data set.

It is striking that most of the active chemicals were in the medium hydrophobicity range. This raises an important question: Are chemicals of medium hydrophobicity more biologically active, or is this simply a result of such compounds being easier to test? We cannot answer this question with the presently available experimental data. Not only in ToxCast/Tox21 but also other large-scale initiatives such as the Precisiontox project,^83^ data availability is skewed toward easy-to-test chemicals and intentionally potent chemicals.

For 70% of the cytotoxic chemicals (*n* = 694) a baseline toxicity prediction could be performed and 55% of the cytotoxic chemicals were specifically acting with TR > 10 (Figure 3c), but the TR rarely exceeded TR > 1000. More chemicals activated the oxidative stress response than were cytotoxic (Figure 3a), and they were slightly more hydrophilic than the cytotoxic chemicals (Figure 3b). Only 9% (*n* = 322) of the active chemicals were specific with SR_cytotoxicity_ > 10. Evidently, the number of chemicals with SR_baseline_ > 10 is the sum of the chemicals with TR > 10 and SR_cytotoxicity_ > 10 (Figure 3c). This analysis demonstrates that the majority (91%) of chemicals triggering the oxidative stress response are also cytotoxic, and their hazard would have been identified already with a simple cytotoxicity assay. It could be expected that industrial chemicals have even lower TRs and SRs because the chemical sets of ToxCast and Tox21 are rich in intentionally potent chemicals.

An SR_baseline_ close to 1 does not mean that the specific effect is irrelevant, but it means that it is predictable by the baseline toxicity model, which greatly simplifies hazard assessment. This is important as the example of per- and polyfluoroalkyl substances (PFAS) shows. In thousands of studies on the toxicity, PFAS will trigger almost every type of effect, albeit often at high concentrations.^85^ Why is that? The carboxylic acids (e.g., PFOA) and sulfonic acids (e.g., PFOS) bind strongly to proteins and act as surfactants. Thus, they can disturb any enzyme or receptor and will damage any membrane, to begin with cell membranes but also more specialized membranes such as mitochondrial membranes or axons and dendrites of neurons. When analyzing the SR of PFAS in several NAMs, it turned out that most PFAS were active in many bioassays but essentially triggered these diverse effects at baseline toxic concentrations.^86^ It is important enough to repeat: baseline toxicity does not equal low toxicity. However, the charm of baseline toxicity is that it is easily predictable with a simple mechanistic model (Figure 2).

## Are In Vitro Data Ready for Prediction Models?

NAMs also include in silico models. Traditional QSAR models for prediction of toxicity in vitro and in vivo with clearly defined applicability domains have evolved in the past decade into machine learning (ML),^87^ then deep learning (DL),^88^ and finally artificial intelligence (AI)^89^ models. As present version models are often focused on the technical quality of the algorithms, the quality of the input data is rarely sufficiently scrutinized, and dose–response data are typically converted into categorical data despite “no effect detected” not meaning “not toxic” as outlined above. Experimental artifacts propagate in these models to false predictions that are not recognized as such.

Many of the currently available in silico models were trained with the ToxCast and Tox21 10K library,^90^ which in principle deliver dose–response data but model inputs were in the past often simplified to categorical (yes/no) data.^91–94^ As argued above, toxicity is never simply categorical. Even specific receptor-binding effects can only be assigned as “not active up to the concentration where cytotoxicity kicks in”, and a lot of the chemicals assigned as inactive are classified as such because they were not active within the tested concentration range.

Recently ML models were expanded to prediction of quantitative toxicity estimates,^95^ but there remains the problem of missing data that are not true inactive but missing due to inadequate experimental planning or experimental difficulties. We suggest filling data gaps by baseline toxicity, which can be easily predicted from physicochemical properties as shown in Figure 2. While baseline toxicity does not provide conservative estimates, they are typically within 2–3 orders of magnitude of experimental data.^76^

Big data models should be transitioned from categorical to dose–response data to avoid the existing problem of categorical toxicity data, but ultimately, the experimental database of robust NAM data needs to be further expanded to larger numbers of industrial chemicals covering a broader physicochemical space of current-use chemicals to make these models more robust.

## NAMs for Comparative Hazard Assessment

NAMs offer an untapped opportunity for the assessment of replacement products and the design of SSbD chemicals. Any chemical that becomes restricted under REACH has undergone several years of in-depth assessment. A prominent example is bisphenol A (BPA), which was phased out as an industrial chemical in REACH due to its endocrine-disrupting properties. It took years and the sacrifice of hundreds of test animals to collect enough evidence to restrict the use of BPA on the European market. This gave enough time for development and marketing of dozens of replacement products, some of which have now also drawn attention to their hazard but many are used without any restriction despite their striking similarities.^96^ Legally, any replacement product undergoes the same evaluation process as BPA and can be marketed for the time being, which has led to increased concentrations of BPA alternatives detected in the environment^97^ and humans.^98^ Many BPA alternatives are structurally very similar to BPA and have a similar NAM-based toxicity profile.^96,99^ Srebny et al.^99^ proposed using a combination of relative potency compared to BPA as well specificity ratio scores across diverse toxicity end points to rank BPA alternatives and demonstrated that all alternatives with a bisphenol core structure were regrettable substitutions and only a few were marginal improvements. Only alternatives without the bisphenol core structure had a better hazard profile. This study may be a blueprint for future comparative hazard assessment studies.

## Persistent Toxicity as Novel Hazard Indicator

NAMs are by definition short-term assays but can provide conclusions about long-term toxicological hazards if MIE and KE relevant for chronic toxicity are assessed. Persistence is in itself an important hazard indicator^100,101^ but has also important implications for toxicity. Chemicals that are persistent will reside longer in the environment and are eliminated more slowly from the human body once taken up. Thus, chronic exposure is more likely for persistent chemicals.^6^ Even if uses of persistent chemicals are phased out, these chemicals will persist for decades in the environment and be spread throughout, as we have learned from the examples of persistent organic pollutants (POPs) such as organochlorine chemicals, dioxin-like chemicals, and polychlorinated biphenyls.^100^ What one can further learn from the history of POPs is that we have not learned our lesson about the key importance of persistence. Instead, organochlorine chemistry was followed by organobromine chemistry, with organobromine compounds regularly used as flame retardants and many other functions until they were also classified as POPs.^102^ It is now increasingly recognized that even persistent and mobile chemicals can pose a hazard despite their oftentimes low toxicity.^103,104^

PFAS are the ultimate persistent chemical class and have even been termed “forever chemicals” for their nearly unbreakable C–F bond in the natural environment.^105,106^ Many of them have surfactant-like properties, and the anionic PFAS have high specific binding affinities to proteins. PFOS and PFOA are archetypes of promiscuous molecules. Even without invoking any highly specific effect, they will provoke effects wherever they interact with biomolecules in a cell. As hydrophobic surfactants, they will disturb the structure and function of biomembranes, leading to mitochondrial toxicity and neurotoxicity. As anions, they will disturb many different protein functions and receptors and will bind especially strongly to and accordingly disturb receptors for fatty acids. We can foresee that they will continue to do so for a long time to come because even if we phased out production of all chemicals as of today, PFAS would still remain in human bodies and in other biota, being slowly depurated but continuously taken up again through food and water. In summary, PFAS do not need a high and specific toxicity to make them hazardous chemicals, but it is sufficient for them to be persistent and cause effects with low SR but for many different end points to be chemicals of very high concern.

Besides highly persistent chemicals, there have also been instances of chemicals that degrade but, in doing so, form highly hazardous transformation products. To capture both, the persistent and toxic substances as well as those that degrade but form hazardous TPs, we have earlier proposed a concept to link toxicity and persistence assessment using high-throughput NAMs.^107^ This concept is based on assessing each chemical in terms of two hazard indicators, called Cumulative Toxicity Equivalents (CTEs) and Persistent Toxicity Equivalents (PTEs). We have suggested that this concept could simplify hazard assessment and consolidate PBT indicators with the emerging PMT indicators.^107^

The proposed “Toxicity Equivalents” can be measured exclusively with animal-free, high-throughput in vitro bioassays. CTE refers to the toxic effects measured directly in any given sample, including single chemicals, substitution chemicals, or products.

PTE is the equivalent measure of CTE measured after the simulated environmental degradation of the sample. PTE does not require analytical identification of transformation products (TP) but will account for the contribution of TPs to effects by TPs. While conceptually convincing, the practicality of linking biodegradation with toxicity measurements poses several challenges. A high-throughput miniaturized biotransformation assay using sewage sludge in small volumes was recently developed that allows the robust measurement of environmentally relevant biodegradation rate constants in only 48 h because of the higher bacterial density than in the OECD biodegradation simulation tests.^108,109^ For PTE, the concentrations in the biodegradation must not be so high to damage or alter the bacterial community, but then the resulting reaction mixtures need to be enriched to a degree that effects can be detectable by NAMs. For optimizing the enrichment step it must be considered that transformation products are typically more hydrophilic, which means that the extraction should combine extraction with a solvent and solid-phase extraction that also captures charged and hydrophilic chemicals.^110^ The criterion to be defined is that if the effect decreases to a certain level (e.g., 50% of the initial effect) within 48 h, then there is no persistent toxicity. If this is not the case, then it must be investigated whether the parent compound is persistent by comparing the measured effect with the change in the concentration of the parent compound. If the toxicity is not proportional or even increasing, then (potent) TPs are formed.^111^ Experimental approaches are presently under development and will be presented soon.

For SSDB one could go a step simpler especially during the design phase when no or only small amounts of test chemicals are available, and the coupling of biodegradation with toxicity assessment is experimentally challenging because one has to find concentration ranges that are nontoxic to the degrading bacteria but then enrich enough to detect toxicity after degradation. Alternatively, one could tackle persistence using simple high-throughput chemoassays that simulate toxicokinetics and degradability.^112^ Toxicokinetics can be simulated with a biomimetic aCYP catalyst^113^ and directly be integrated in NAM toxicity assays.^99^ A battery of well-plate-based assays including hydrolysis, reactivity toward glutathione and proteins, and photodegradation was developed to quantify abiotic stability of chemicals in in vitro bioassays^112^ but may well be expanded and used for persistence assessment by reaction with hydroxy radicals (·OH) or other strong oxidants.

## Outlook

The development of NAMs, test battery development, and validation are moving with a fast pace,^3^ but some hurdles still need to be overcome before they can be confidently applied in the hazard assessment of industrial chemicals. Most importantly, effect concentration thresholds for different hazard categories must be developed by competent authorities. These categories should be harmonized with the hazard categories in the Globally Harmonized System for Classification and Labeling of Chemicals (GHS).^114^

The lack of testing of very hydrophobic chemicals as well as insufficiently broad dosing ranges has led to classification artifacts that potentiate whenever these incorrect classifications are used to train ML and AI hazard prediction models. For the time being, one can substitute missing data with baseline toxicity predictions, which is not precautionary but better than classifying difficult chemicals as nontoxic. Even existing OECD guidelines for NAMs^115–118^ have been typically validated with easy-to-test and highly specific (intentionally potent) chemicals. These NAMs are often not suitable for testing difficult chemicals, but lessons can be learned from the OECD guidance document on aqueous-phase aquatic toxicity testing of difficult test chemicals,^119^ which will hopefully be implemented in future revision of the GD34 on validation and acceptance of NAMs.^120^

If a chemical is persistent and has a log *K*_ow_ exceeding a certain threshold, it is hazardous and will ultimately contribute to mixture toxicity in real life even if it is not bioaccumulative in the time frame of standard experiments or if no effect is detected in a standard animal assay or a NAM up to the highest tested concentration. We do not need to invoke a specific effect for a very hydrophobic chemical to be deemed hazardous if it is at the same time persistent. It is different at the opposite site of the hydrophobicity scale: Hydrophilic and charged chemicals are not highly bioaccumulative, and they will only be classified as hazardous if they exhibit specific modes of action or even reactive modes of action (which many of them do). If we accept this chemical perspective on toxicity, we are a step closer to safer chemicals and can avoid regrettable substitutions in the future.

## Glossary

ACC: activity concentration at cutoff (benchmark value used in toxicology to identify the concentration of a chemical at which a measured biological effect first exceeds the unexposed control)
AC50: median activity concentration in in vitro bioassays
aCYP: abiotic cytochrome P450 (CYP) enzyme (biomimetic catalyst 5,10,15,20-tetrakis­(2,6-dichlorophenyl)-porphyrin-Mn­(III))
AI: artificial intelligence (simulation of human intelligence in algorithms that are programmed to think and learn like humans)
ANT: adult neurotoxicity
AO: adverse outcome (final step in the AOP)
AOP: adverse outcome pathway (describes a toxicity pathway)
Baseline toxicity: minimal toxicity of any chemical (also called narcosis)
BPA: bisphenol A
CLP: Classification, Labeling and Packaging (European Union Regulation)
CMB: critical membrane burden (constant concentration in cell membranes that triggers baseline toxicity)
CTE: cumulative toxicity equivalents
DA: Defined Approach (OECD approach to include NAMs in effect assessment)
DL: deep learning (subfield of ML that uses multilayered neural networks to mimic the human brain’s structure for analyzing complex data)
*D*_lip/w_: liposome–water distribution ratios (measure for the hydrophobicity and affinity to biological membranes of an organic chemical)
DNT: developmental neurotoxicity
ECHA: European Chemicals Agency
GHS: Globally Harmonized System for Classification and Labeling of Chemicals (international system that standardizes how chemical hazards are classified developed by the UN)
KE: key event (step in the AOP)
*K*_ow_: octanol–water partition constant (measure for the hydrophobicity of an organic chemical)
MIE: molecular initiating event (step in the AOP)
ML: machine learning (algorithms to enable systems to learn from data and improve over time without being explicitly programmed)
NAM: New Approach Methodologies (in vitro or in silico tools for toxicity assessment without animal testing)
OECD: Organisation for Economic Co-operation and Development
PARC: EU Partnership for the Assessment of Risk from Chemicals (EU research and innovation initiative that aims to improve chemical risk assessment)
PTE: Persistent Toxicity Equivalents (measure of the remaining toxicity of a chemical after it has been subjected to simulated environmental degradation)
QIVIVE: quantitative in vitro to in vivo extrapolation (computational method that uses data from cell-based (in vitro) lab tests to predict toxicity and other effects in a living organism (in vivo))
QSAR: Quantitative Structure–Activity Relationship (computational method that uses statistical analysis to correlate a molecule’s chemical structure with its biological activity)
POP: persistent organic pollutant (toxic, human-made chemicals that persist in the environment, travel long distances, and accumulate in the food chain)
PFAS: Poly- and perfluoroalkyl substances
REACH: Registration, Evaluation, Authorization and Restriction of Chemicals (European Union Regulation)
Regrettable substitution: practice of replacing a harmful chemical with another chemical that is later found to be equally or more hazardous
SSbD: safe and sustainable by design (voluntary European Commission framework for developing or redesigning chemicals and materials to be inherently safer and more sustainable throughout their entire lifecycle)
SMPD: serum-mediated passive dosing (technique for controlled dosing in in vitro bioassays)
SR: specificity ratio (measure of how specific an effect is compared to cytotoxicity or baseline toxicity)
ToxCast: US EPA Toxicity Forecaster (provides accessible bioactivity data for toxicology)
Tox21: Toxicology in the 21st Century (US federal research collaboration focused on developing methods to evaluate chemicals’ safety)
TP: transformation products (new substances formed when a parent compound undergoes chemical reactions through processes like metabolism, environmental breakdown, or other chemical changes)
TR: toxic ratio (measure of enhanced cytotoxicity over baseline toxicity)
